# Longitudinal Validity and Reliability of Brief Smartphone Self-Monitoring of Diet, Stress, and Physical Activity in a Diverse Sample of Mothers

**DOI:** 10.2196/mhealth.9378

**Published:** 2018-09-21

**Authors:** Dallas Swendeman, Warren Scott Comulada, Maryann Koussa, Carol M Worthman, Deborah Estrin, Mary Jane Rotheram-Borus, Nithya Ramanathan

**Affiliations:** 1 Department of Psychiatry and Biobehavioral Sciences David Geffon School of Medicine University of California, Los Angeles Los Angeles, CA United States; 2 Department of Anthropology Emory University Atlanta, GA United States; 3 Cornell Tech Cornell University New York, NY United States; 4 Department of Computer Science University of California, Los Angeles Los Angeles, CA United States

**Keywords:** self-monitoring, mHealth, diet, physical activity, stress, multi-method, mobile phones, C-reactive protein

## Abstract

**Background:**

Multiple strategies can be used when self-monitoring diet, physical activity, and perceived stress, but no gold standards are available. Although self-monitoring is a core element of self-management and behavior change, the success of mHealth behavioral tools depends on their validity and reliability, which lack evidence. African American and Latina mothers in the United States are high-priority populations for apps that can be used for self-monitoring of diet, physical activity, and stress because the body mass index (BMI) of mothers typically increases for several years after childbirth and the risks of obesity and its’ sequelae diseases are elevated among minority populations.

**Objective:**

To examine the intermethod reliability and concurrent validity of smartphone-based self-monitoring via ecological momentary assessments (EMAs) and use of daily diaries for diet, stress, and physical activity compared with brief recall measures, anthropometric biomeasures, and bloodspot biomarkers.

**Methods:**

A purposive sample (n=42) of primarily African American (16/42, 39%) and Latina (18/42, 44%) mothers was assigned Android smartphones for using Ohmage apps to self-monitor diet, perceived stress, and physical activity over 6 months. Participants were assessed at 3- and 6-month follow-ups. Recall measures included brief food frequency screeners, physical activity assessments adapted from the National Health and Nutrition Examination Survey, and the nine-item psychological stress measure. Anthropometric biomeasures included BMI, body fat, waist circumference, and blood pressure. Bloodspot assays for Epstein–Barr virus and C-reactive protein were used as systemic load and stress biomarkers. EMAs and daily diary questions assessed perceived quality and quantity of meals, perceived stress levels, and moderate, vigorous, and light physical activity. Units of analysis were follow-up assessments (n=29 to n=45 depending on the domain) of the participants (n=29 with sufficient data for analyses). Correlations, R^2^ statistics, and multivariate linear regressions were used to assess the strength of associations between variables.

**Results:**

Almost all participants (39/42, 93%) completed the study. Intermethod reliability between smartphone-based EMAs and diary reports and their corresponding recall reports was highest for stress and diet; correlations ranged from .27 to .52 (*P*<.05). However, it was unexpectedly low for physical activity; no significant associations were observed. Concurrent validity was demonstrated for diet EMAs and diary reports on systolic blood pressure (r=−.32), C-reactive protein level (r=−.34), and moderate and vigorous physical activity recalls (r=.35 to.48), suggesting a covariation between healthy diet and physical activity behaviors. EMAs and diary reports on stress were not associated with Epstein–Barr virus and C-reactive protein level. Diary reports on moderate and vigorous physical activity were negatively associated with BMI and body fat (r=−.35 to −.44, *P*<.05).

**Conclusions:**

Brief smartphone-based EMA use may be valid and reliable for long-term self-monitoring of diet, stress, and physical activity. Lack of intermethod reliability for physical activity measures is consistent with prior research, warranting more research on the efficacy of smartphone-based self-monitoring of self-management and behavior change support.

## Introduction

### Background

Smartphones are increasingly used and integrated into daily routines, creating opportunities for continuous, real-time data streams of health behaviors and states [1,2]. Such data streams include self-reports, such as ecological momentary assessments (EMAs) and daily diaries [3]. The reliability and validity of smartphone apps for self-monitoring health behaviors is not yet fully understood but is critical for generating an evidence on the growing field of mobile health or “mHealth” [1] and for its broad adoption by consumers [4].

Diet, stress, and physical activity are the key lifestyle factors associated with a broad range of physical and mental health issues, such as obesity, diabetes, cardiovascular disease, depression, and anxiety [5]; for example, cardiovascular disease is a significant health problem that is still largely ignored by women, especially young women [6], although it accounts for a higher mortality rate than all forms of cancer in women [7]. Mothers are included in a high-priority target population because their body mass index (BMI) typically increases by 5 kg for several years after giving birth [8].

Smartphones are well suited for real-time self-monitoring using daily diaries and more frequent EMAs of diet, perceived stress, and physical activity because these behaviors and states can be difficult to recall precisely over longer periods of time and can vary significantly within and across days [3]. Smartphone-based EMA and diaries of target behaviors may be more specific and feasible than biomarkers and biomeasures (eg, BMI and blood pressure), which typically reflect the accumulated impact of several factors on physiological systems over time. Active self-monitoring (ie, via self-report) is also an important behavior change technique [9,10], particularly for the self-management of diet and physical activity [11]. Self-monitoring is nearly a universal behavior change element in smartphone apps for diet and physical activity self-management [12-14].

Smartphones lighten the burden of one aspect of EMAs by allowing data entry on a readily available device that is close at hand; cumbersome paper diaries or personal digital assistants of yesteryears are no longer needed. The intensity of EMA that requires daily reporting at various time-points throughout the day remains. Not surprisingly, decreases in adherence to mobile phone-reported EMAs over time have been noted for disparate outcomes, including nutrition, mood, and use of substance measures [15,16]. Therefore, it is important to determine which measures that need to be captured via EMAs and those that can be captured less frequently. Such an assessment has been a challenge due to limited validity and reliability studies in mobile phone-reported diet, stress, and physical activity measures, which is the focus of this study because there are no accepted gold standards for in situ assessments of these behaviors that can be readily used for objective comparison [17-19]. Studies have demonstrated discrepancies between retrospective self-reports and EMAs as well as their benefits and limitations [20-22]. Some studies have compared self-reported health behaviors to more objective measures, such as self-reported physical activity and that obtained using a pedometer [23-26]. In this study, we observed an intermethod reliability between smartphone EMAs and diary reports and their corresponding recall reports in addition to EMAs and recall between health measures, such as diet and exercise, that we anticipate to be correlated to each other. Such studies on ethnic minorities and women are also limited [11,27]. Toward this goal, we examined data from a feasibility study that pilot-tested a health-behavior self-monitoring mobile app in a sample that mostly included ethnic minority mothers; a prior study has examined the predictors of self-monitoring adherence (BLINDED) [28]. This paper examined the validity and reliability of brief use of smartphone-based EMAs and daily diaries for diet, stress, and physical activity compared with those of the brief recall self-reports, simple anthropometric biomeasures (eg, weight and BMI), and laboratory biomarkers (ie, C-reactive protein and Epstein–Barr Virus) collected at 3-month intervals over 6 months. We evaluated the intermethod reliability and concurrent validity of the app, which are high priorities for mobile health, “mHealth,” evidence development [1]. Different fields employ divergent conceptualizations of validity and reliability. However, in this study, intermethod reliability is broadly conceptualized as concurrency between different methods assessing the same domain, whereas concurrent validity is conceptualized as concurrency between the assessments of different but linked domains (ie, diet, stress, and physical activity), and it uses multiple assessment methods that provide information on the concurrent validity of methods.

### Hypotheses

This study assessed several sets of interrelated hypotheses to evaluate the reliability and validity of the brief use of EMAs and diary questions designed for smartphone apps. First, we hypothesized that the brief use of smartphone-based EMAs and daily diary questions would demonstrate intermethod reliability through associations with their corresponding recall self-reports. Second, we hypothesized that the EMAs and daily diary measures would demonstrate concurrent validity through associations with anthropometric biomeasures, bloodspot biomarkers (for stress), and recall and EMAs and diary reports on other domains. Third, given that EMAs are designed to be independent and captured close to or in the moment, we hypothesized that EMAs would be more reliable than daily diaries for diet and perceived stress, which may be difficult to recall due to a high variability throughout the day. A study indicating that daily diary reports are comparable with EMA would suggest that a less burdensome diary method as preferable for future applications. However, we hypothesized that the daily diaries for physical activity would be sufficient to achieve minimal recall biases. Thus, EMAs for physical activity were not assessed to minimize burden.

## Methods

### Participatory Sensing

Development and study procedures were based on a participatory sensing approach used in mobile phone sensing projects developed by computer scientists [29]. User-centered design principles prioritize participant autonomy and choice over which features of the app to use (eg, responding to surveys) and recognize varying ability and motivation to adopt and sustain the activities. Similar to pragmatic designs in implementation research [30], participatory sensing’s emphasis on naturalistic use prioritizes the external validity or generalizability of the sensing tool used across diverse user preferences, participation options, and motivations for participation. In this study, user preferences were collected through focus groups (BLINDED) and iterative trials with participants. The participatory sensing approach was used as a basis for designing the questions for EMA and diary questions which are brief, engaging, and meaningful for self-monitoring using the app for self-management rather than being granular and precise as a gold standard that is more typical in basic behavioral EMA studies.

### Ethics Statement

The institutional review board of the University of California Los Angeles reviewed and approved the study. All participants signed informed consent. The study was conducted in accordance with the Declaration of Helsinki.

### Recruitment and Participants

Mothers residing in an urban area who had at least one child living in the household were recruited to participate in the study from January 2012 to September 2012. Recruitment flyers framed the study as seeking support to develop and pilot-test a smartphone app that can help in the self-monitoring and self-management of diet, stress, and physical activity. Recruitment included weekly visits to local farmer markets; classes and groups at a community center; outreach at local grocery stores, churches, and targeted community organizations; and posting on local online groups regarding parenting and children. Women with a child below 18 years who is living at home, those who were not pregnant or breastfeeding, and those with a BMI ≤18.5 (ie, dangerously underweight) were included in the study. The recruitment plan aimed to include a sample of mothers with diverse BMIs (about one-third were normal in terms of weight, overweight, or obese) and those who were primarily African American and Latina. However, other race or ethnic groups were not excluded.

### Measures

#### Smartphone Ecological Momentary Assessment and Daily Diary

Participants were assigned Samsung Vibrant smartphones and were instructed to complete the smartphone EMAs and daily diary surveys by responding to time-based (ie, alarm) prompts and event-based reports (ie, self-initiated). The participants selected the start and end times for three 3-hour windows for time-based EMA prompts (morning, midday, and late afternoon) and one end-of-day daily diary survey. The first three surveys asked about diet “since the last survey” and stressful events “over the last 2 hours.” The end-of-day “daily diary” survey asked about diet, stress, and physical activity for the entire day. The following variables were used in the analyses:

##### Diet Ecological Momentary Assessment

Three times a day, the participants were asked the following question: “Rate the nutritional quality of this meal?” The responses were as follows: (1) low; (2) medium; and (3) high.

##### Diet Diary

At the end of the day, the participants were asked the following question: “How healthy would you rate your eating today, in terms of both quality and quantity, on a scale of 1 to 5 (with 5 being very healthy)?”

##### Stress Ecological Momentary Assessment

Three times a day, the participants were asked the following question: “Have you felt stressed in the last two hours?” The responses were as follows: (1) not at all, (2) slightly, (3) moderately, and (4) very.

##### Stress Diary:

At the end of the day, the participants were asked the following question: “How stressful was your day overall on a scale of 1-5 (with 5 being very stressful)?”

##### Physical Activity Diary

At the end of the day only, three questions were asked: “How many minutes of activity did you do today.” The responses were as follows: for three intensities of physical activity, light (ie, “no increase in breathing or heart rate, eg, stretching”), moderate (ie, “small increase in breathing or hearth rate, eg, fast walking”), and vigorous (“significant increase in breathing or heart rate, eg, running”). To compare the recall reports for each activity type, the variables were calculated as number of days (over 30 days), minutes per day on average, and total minutes (minutes × days).

#### Baseline and Follow-up Recall Self-Reports

Measures were brief and reflective of the scope and scale of measures that are likely to be used in clinical practice or large-scale survey research. Recall periods were retained from the original measures, which were designed to minimize recall biases and capture general habits rather than detailed, gold-standard assessments.

##### Demographics Characteristics of the Participants

The background factors assessed included age, race or ethnicity, highest level of education, work hours per week (paid, volunteer, or student), and number of children living in the home.

##### Dietary Behaviors

Food frequency questionnaires from the California Health Interview Survey (CHIS) were used to assess diet; CHIS 2009 Adult questionnaire ver 3.4 (Public) March 1, 2011, Section C, p 32. These brief screening measures have a validity sufficient to discriminate higher or lower intakes among individuals, particularly for examining the relationships between diet and other variables, and they were used by the Applied Research Program in the Division of Cancer Control and Population Sciences at the National Cancer Institute for diet screening. Ten questions assessed the number of times a participant ate or drank various foods over the past 30 days, including prompts to estimate times per day, week, or month. Food types were categorized into three variables in the analyses: intake of fruits and vegetables (three questions on fruits, green vegetables or salad or beans, and nonfried potatoes), intake of food with high-sugar content (four questions on sugar-rich drinks or soda, sweetened fruit drinks, cookies, cake, and ice cream), and intake of fast food (one question over the past 7 days).

##### Perceived Stress

The brief, nine-item psychological stress measure (PSM-9), which is designed to assess for stress in primary care settings, was used [31]. The PSM-9 was developed from an original 49-item version and then two 25-item versions, which showed a high internal consistency (Cronbach alpha coefficients of 0.92 and 0.93) and test-retest reliability (0.68-0.80). Convergent, divergent, concomitant, and predictive validity were established by comparing the measures of various constructs, such as depression and anxiety [31]. PSM-9 questions were established according to both related and redundant contents from the longer item versions, covering domains, such as feeling calm, stressed, rushed, worried, confused, and energetic, physical symptoms, and difficulty controlling reactions or emotions.

##### Physical Activity

Physical activity was assessed using the questions from the CHIS 2009 survey that ask about the number of times per week (within the last 7 days) and average minutes per day for walking (for transport and recreation or physical activity), moderate activity (ie, breathe somewhat harder than normal), and vigorous activity (eg, aerobic sports, breathe significantly harder than normal). These questions are similar to those used in the National Health and Nutrition Examination Survey and other studies [17]. The two walking domains were combined, and the following variables were used for the analyses: walking (minutes per day, number of days, and total minutes), moderate physical activity (minutes per day, number of days, and total minutes), vigorous physical activity (minutes per day, number of days, and total minutes).

#### Biomeasures and Biomarkers

The biomeasures and biomarkers used in this study were selected for meeting the criteria on feasibility, acceptability, minimal invasiveness, and the comprehensive indicators of diet, stress, and physical activity. Simple anthropometric measures (height and weight to determine BMI, body fat measurements, and waist circumference) were used as indicators of physical activity [17]. Blood pressure was used as an indicator of overall health, which includes factors correlated to diet, physical activity, and stress and stressors [32,33]. Bloodspot C-reactive protein and Epstein–Barr virus antibodies were used as proxy measures of stress that respectively identified inflammation and cardiovascular risk and allostatic load correlated to a variety of stressors and stress-related impairment of innate immune capacity; this result is similar to that of prior research on maternal stress [34]. Although low-grade inflammation and weakened innate immunity have been linked to lifestyle and psychosocial factors, such as overnutrition, depression, and obesity [35-38], in the literature, stress is identified as a common cause of inflammation and alterations in innate immunity [39-41]. C-reactive protein and Epstein–Barr virus antibodies have been positively associated with stress, and they are often used as biomarkers of stress in research, as well as in this study [42-44]. Body fat was measured with the Tanita body composition analyzer (Model Tbf-300a) that uses bioelectrical impedance analysis, a commonly used method for estimating body composition [45,46]. Because fluctuations in hydration may affect body composition results, the participants were asked to avoid diuretics for 7 days, alcohol for 48 hours prior, intense physical activity for 12 hours prior, eating or drinking for 4 hours prior, emptying of bladder 30 minutes prior to assessment, and rescheduling if they became ill.

##### Body Mass Index

Height was measured in meters using a portable stadiometer, and the Tanita body composition analyzer scale was also used to measure weight in kilograms. Company and model information for the stadiometer is unknown. BMI was calculated as weight in kilograms divided by height in meters squared.

##### Waist Circumference

Waist measurements were taken using standardized procedures recommended in the Anthropometric Standardized Reference Manual [47]. The measuring tape was placed around the waist area (midway between the rib cage and hip bone). It was ensured that the tape was snug without compressing the skin and was parallel to the floor. Measurements were taken after the participant had exhaled, and the average of the three separate measurements was obtained.

##### Blood Pressure

Systolic and diastolic blood pressure were measured at the midpoint of the upper arm using an Omron automatic blood pressure monitor (HEM-705CP). The participants were asked to be seated with their back supported and legs uncrossed and were instructed to support their arm on a table so that the midpoint of their upper arm was at the level of the heart. Three readings were obtained. The first reading was discarded, and the average was taken using the final two readings [48].

##### Bloodspot Tests

A single finger stick from a microlancet (Becton-Dickson Microtainer contact-activated lancet, high blood flow 366594) was used to collect 5 drops of blood and place them onto the preprinted filter paper card (250 μL), which is commonly used for neonatal screening and does not require immediate freezing [49]. Samples were labeled, dried for 4 hours, and stored in a plastic container in a locked refrigerator. Bloodspot samples were shipped each week to the laboratory and stored at −28°C until analysis for C-reactive protein and Epstein–Barr virus.

##### C-Reactive Protein

We used a validated biotin-streptavidin immunofluorometric assay for bloodspot C-reactive protein level, as reported elsewhere [50]. Based on the methods outlined herein [50], we developed an algorithm for serum equivalent values: serum (C-reactive protein value)=1.7*(bloodspot C-reactive protein value). The values indicate the extent of chronic low-grade systemic inflammation associated with cardiovascular and metabolic risk [51]. The C-reactive protein values were classified as low (<1 mg/L), medium (1-3 mg/L), and high risk (>3 mg/L; [52,53]). Values >10 mg/L indicate acute inflammation, and they were not used in the statistical analyses.

##### Epstein–Barr Virus Antibodies

Epstein–Barr virus antibody titers reflect the degree of immune response impairment. The enzyme-linked immunosorbent assay (ELISA) assay used for Epstein–Barr virus antibodies in blood spots is a modification of a commercially available kit (Number P001606A; DiaSorin Corporation, Stillwater, MN); method and validity have been reported elsewhere [49,54]. Antibody titers were presented as ELISA units. Values <20 indicate undetectable antibody levels [54], and they were not used in the statistical analyses. In the absence of standard health-risk categories for Epstein–Barr virus, we compared the mean levels of our sample to those from other samples obtained mostly from ethnic minority women in Illinois (n=183; mean Epstein–Barr virus level=136.8; [34])

### Design

The participants (N=56) were randomly categorized into two groups. The experimental group (n=44) was asked to self-monitor their condition using Android smartphones with the self-monitoring app. The control group (n=12) was not provided with smartphones. The primary aim of the study was to assess the validity and reliability of using smartphones in measuring diet, stress, and physical activity. The control group was designed for secondary, preliminary efficacy aims not reported in this paper, and it did not show statistically significance in the preliminary analyses. Retrospective assessments and biomarker collection were conducted at baseline as well as 3 and 6 months after enrollment to estimate the time between clinical visits for moderately acute ill patients. The participants assigned to the smartphone group were instructed to use the app over the 6-month study period. Two of the 44 participants in the self-monitoring group got pregnant within 2 months after enrollment, dropped out of the study, and were excluded from the sample. The units of analysis were 3-month follow-up during the 6-month study period for 42 participants (n=84 possible units, two periods for each participant).

### Procedure

*Ohmage: Smartphone App.* Ohmage is a mobile to web platform that supports the collection, storage, analysis, and visualization of self-report and passive sensor data streams. Ohmage has been released as an open source, and it can be downloaded for free online. The platform is feature-rich and extensible, and facilitates the collection of multidimensional, heterogeneous, and complex personal data streams. Ohmage adds a time and location stamp to each data point. Web interfaces are available for researchers to access and view data. The Ohmage user interface was designed based on expert feedback from behavioral science collaborators and nonexpert pilot users through focus group (BLINDED) and one-on-one interviews during a pilot phase. Notably, most of the discussions with the participants focused on reducing the user burden when self-reporting data.

#### Incentives

At the end of the study, upon returning the assigned study equipment and completion of the final assessment, the participants received reimbursement for parking and transportation and cash or a gift certificate up to US $355 for completing the following: US $40 each for baseline, 3-month, and 6-month follow-up assessments with biomarkers and biomeasures and US $1.30/day for completing at least one EMA or diary survey per day for 6 months (approximately 180 days).

### Analyses

Prior to analysis, EMA and diary measures were averaged during a 30-day period that ended on and included the date of recall assessment and biomarker measurement. Thirty-day periods were chosen to broadly capture the time frame imposed by the recall measures. Analyses exclude 30-day periods with less than 14 days of EMAs or diary reports (ie, less than 50% reporting) for each specific comparison with a recall or biomarker measure.

The primary analytic goal was to examine the relationship between pairs of measurements, where the first measurement is an EMA or diary measure, aggregated over 30-day periods, and the second measurement was a recall or biomarker or biomeasure. To fully explore meaningful relationships between measurement pairs, several metrics of association that address both clinical significance (eg, correlations) and statistical significance (ie, *P* values) were presented.

*Pearson product-moment correlations* were calculated using measurement pairs as a basic measure of association. To calculate *P* values for the statistical significance of the correlation between measurement pairs, random effects linear regression models were utilized for correlations between measurements in the same individual, and they were expressed as follows:

Y_ij_ = β_0_+ X_ij_β_1_+ λ_i_+ ε_ij_

Where Y_ij_ is an EMA measurement for participant i at time point j, β_0_ is an intercept term, and β_1_ is a regression coefficient for X_ij_, either a recall or biomarker measurement and represents the association within measurement pairs, which is similar to the correlation. Participant-level random effect λ_i_ identifies the correlation between repeated measurements in the same individual, and ε_ij_ are the residual error terms. *P* values were presented for the significance of β_1_.

**Table 1:** Linear relationships between the pairs of ecological momentary assessments or daily diary responses.

Ecological momentary assessments and diary response predictor		n	r^a^	*P* value^b^	R^2^_Ed_^c,d^	R^2^_Total_^e^
**Diet ecological momentary assessments (3 times per day)**						
	Recall: Intake of fruits and vegetables	36	0.34^f^	0.006	0.23	0.05
	Recall: Intake of food with high-sugar content	34	−0.52^f^	0.004	0.27	0.24
	Recall: Intake of fast food	33	−0.42^g^	.02	0.17	0.18
	Recall: Moderate physical activity (min/day)	35	0.48^g^	.03	0.17	0.19
	Biomarker variable: Body fat	38	−0.33	.07	0.13	0.07
	Biomarker variable: Systolic blood pressure	38	−0.32^g^	.02	0.14	0.08
	Biomarker variable: Diastolic blood pressure	38	−0.26	.10	0.08	0.05
	Biomarker variable: C-reactive protein level	35	−0.34^g^	.02	0.16	0.11
**Diet diary**						
	Recall: Intake of fruits and vegetables	30	0.27	.09	0.10	0.07
	Recall: Intake of food with high-sugar content	29	−0.40^g^	.04	0.16	0.14
	Recall: Moderate physical activity (min/day)	31	0.45	.09	0.11	0.14
	Recall: Vigorous physical activity (min/day)	31	0.47^g^	.02	0.34	0.17
	Recall: Vigorous physical activity (total min)	30	0.35^f^	.01	0.45	0.11
**Stress ecological momentary assessments (3 times per day)**						
	Recall: PSM-9^h^ (stress inventory)	45	0.30^g^	.01	0.27	0.08
	Recall: Walking (min/day)	42	0.27^g^	.05	0.18	0.06
**Stress diary**						
	Recall: PSM-9 (stress inventory)	31	0.50^g^	.02	0.28	0.24
	Recall: Walking (min/day)	30	0.38^g^	.05	0.14	0.18
	Recall: Walking (total min)	30	0.36	.06	0.14	0.15
	Biomarker variable: C-reactive protein level	29	−0.28	.09	0.12	0.04
**Light physical activity diary (days)**						
	Recall: Walking (days)	31	0.16	.26	0.05	−0.01
	Recall: Moderate physical activity (days)	31	0.43^9^	.04	0.15	0.16
**Moderate physical activity diary (days)**						
	Recall: Moderate physical activity (days)	31	0.33	.07	0.11	0.08
	Biomarker variable: Body mass index	32	−0.40^g^	.02	0.16	0.13
	Biomarker variable: Body fat	32	−0.35	.06	0.18	0.09
	Biomarker variable: Systolic blood pressure	32	−0.24	.20	0.06	0.03
	B: Diastolic blood pressure	32	−0.34	.07	0.13	0.09
**Vigorous physical activity diary (days)**						
	Recall: Vigorous physical activity (days)	30	0.34	.07	0.11	0.09
	Biomarker variable: Body mass index	32	−0.44^g^	.02	0.26	0.16
	Biomarker variable: Body fat	32	0.44^g^	.02	0.25	0.16
	Biomarker variable: Systolic blood pressure	32	−0.25	.22	0.05	0.03
	Biomarker variable: Diastolic blood pressure	32	−0.35	.07	0.12	0.09
**Vigorous physical activity diary (min/day)**						
	Recall: Vigorous physical activity (min/day)	31	0.27	.30	0.04	0.03
	Biomarker variable: Body mass index	32	−0.38	.06	0.17	0.10
	Biomarker variable: Body fat	32	−0.37	.07	0.15	0.09
**Vigorous physical activity diary (total min)**						
	Recall: Vigorous physical activity (total min)	30	0.35	.11	0.10	0.09
	Biomarker variable: Body mass index	32	−0.37	.07	0.15	0.09
	Biomarker variable: Body fat	32	−0.35	.09	0.13	0.07

^a^r: Pearson product-moment correlations.

^b^*P* values for regression coefficients.

^c^R^2^ statistics based on random effects linear regression models.

^d^Based on the calculation of Edwards et al (2008).

^e^Based on the ratio of unexplained-to-total variance.

^f^*P* value≤.01.

^g^*P* value≤.05.

^h^PSM-9: nine-item psychological stress measure.

Lastly, R^2^ statistics that describe the amount of variation explained by the model were presented. There is no standard R^2^ calculation for the random effects models. Two reasonable formulations were presented. Edwards et al [55] have calculated R^2^ for regression coefficients as follows:

R^2^_Ed_ = (q-1)v^-1^F(β*,Σ*)/[1 + (q-1)v^-1^F(β*,Σ*)]

Where q-1 is the number of regression coefficients minus the intercept term, v is the residual degrees of freedom based on the Kenward-Rogers approximation, and F(β*,Σ*) is the F statistic that is used to test the null hypothesis stating that q-1 regression coefficients are equal to 0. Models were fit using the PROC MIXED procedure in SAS software version 9.3 (SAS Institute Inc, Cary, NC). The second R^2^ statistics was based on the total variation explained and was expressed as follows:

R^2^_Total_ = (Var_int_ – Var_full_) / Var_int_

Where Var_int_ is the total variation from a model that contains an intercept term, random effects, and a residual error term and Var_full_ is the total variation from the full model with the intercept term and all covariates.

#### Multivariate Analyses

As a secondary analytic goal, multiple-predictor linear regression models that regress EMAs and diary measurements on recall and biomarker measurements were established to examine the significant associations, and they further provided insights about the reliability and validity of the EMAs and diary measures. The models were summarized as follows: recall and biomarker→EMA. The candidate predictors for the multiple-predictor regression models include all the variables shown in Table 1, and they were selected based on the four statistics that were presented for the primary analyses: Pearson correlation coefficients, *P* values, β1, and two R^2^ statistics. We aimed to strike a balance between parsimony and prediction in our model. Therefore, *P* values ≤.10 were roughly considered for the entry of predictors into the multiple-predictor models as well as the direct corresponding recall measure regardless of *P* value. Moreover, we wanted to achieve reasonable levels of explained variation based on the R^2^ statistics and strengths of associations based on the Pearson correlation coefficient. Once the multiple-predictor models were built, a backward stepwise selection procedure was used to select predictors for the final models. Predictors were retained at a .05 alpha level.

## Results

### Demographic Characteristics of the Participants

Table 2 and Table 3 present the demographic characteristics of the participants (n=42) assigned with smartphones and those (n=29) with sufficient data for the analysis based on the participation rates (described below). No significant differences were found in terms of the characteristics of the participants who were included (n=29) and excluded (n=13) from the analyses owing to missing data. Most participants self-identified as ethnic minority (85%, 35/41), of which 39% (16/41) identified as African American, 44% (18/41) as Latina, and 2% (1/41) as Other. The age of the participants ranged from 20 to 43 years with an average age of 31.2 years. Approximately one-third of the participants were working full-time or part-time (between 4 and 20 hours a week) or not working. A little over half of the participants were obese or very obese (57%, 24/42) in line with an average body fat percentage of 40%. On average, the participants had blood pressure readings within the normal range (mean systolic blood pressure=122.3 and diastolic blood pressure=79.8). Average C-reactive protein levels of 3.2 mg/L indicated intermediate risk for cardiovascular disease. Average Epstein–Barr virus levels of 140.3 ELISA units were comparable to Epstein–Barr virus levels discussed earlier [34].

**Table 2:** Demographic and baseline biomarker characteristics of mothers who were included and those excluded from the analyses due to low ecological momentary assessments completion rates.

Characteristics		Analysis data (n=29), n (%)	Excluded mothers (n=13), n (%)	Total sample (n=42), n (%)
**Ethnicity^a^**				
	African American	11 (39)	5 (39)	16 (39)
	Asian	2 (7)	0 (0)	2 (5)
	Latina	13 (46)	5 (39)	18 (44)
	White	2 (7)	2 (15)	4 (10)
	Others	0 (0)	1 (8)	1 (2)
**Education^a^**				
	High school or lower	10 (35)	3 (25)	13 (32)
	College	10 (35)	2 (17)	12 (27)
	College degree or higher	9 (31)	9 (58)	16 (39)
**Number of children**				
	1	12 (41)	7 (54)	19 (45)
	2-4	17 (59)	6 (46)	23 (55)
**Body mass index category**				
	Normal weight	5 (17)	2 (15)	7 (17)
	Overweight	8 (28)	3 (23)	11 (26)
	Obese	6 (21)	4 (31)	10 (24)
	Extremely obese	10 (35)	4 (31)	14 (33)

^a^n=41, number of participants excluded due to missing responses.

**Table 3:** Demographic and baseline biomarker characteristics of mothers who were included and those excluded from the analyses due to low ecological momentary assessments completion rates.

Characteristics	Analysis data (n=29), mean (SD)	Excluded mothers (n=13), mean (SD)	Total sample (n=42), mean (SD)
Age	30.9 (6.3)	32.0 (6.0)	31.2 (6.2)
Work hours	22.0 (17.8)	19.5 (20.3)	21.2 (18.4)
Body fat (%)	39.1 (7.6)	40.9 (6.8)	39.6 (7.4)
Systolic blood pressure (mm Hg)	123.3 (15.7)	120.1 (11.0)	122.3 (14.4)
Diastolic blood pressure (mm Hg)	81.3 (9.5)	76.4 (10.0)	79.8 (9.8)
C-reactive protein level (mg/L)^a^	3.2 (2.4)	3.0 (2.6)	3.2 (2.4)
Epstein–Barr virus (enzyme-linked immunosorbent assay units)^b^	135.0 (56.8)	151.8 (67.8)	140.3 (60.2)

^a^n=37, number of participants excluded due to five C-reactice protein values >10.

^b^n=41, number of participants excluded due to one Epstein–Barr virus value <20.

### Participation Rates

Almost all the 42 participants, who used a smartphone, completed the 6-month study (93%; n=39). The analyses excluded 13 participants, including two who were lost to follow-up before the 3-month follow-up, one who moved out of the state before the 6-month follow-up, and 10 participants who had reporting rates <50%.

In total,15,103 time-prompted EMA and diary surveys were completed by 29 participants included in analyses. The responses were distributed uniformly across the four surveys with 4043 (26.8%) morning EMAs, 3855 (25.5%) midday EMAs, 3643 (24.1%) late afternoon EMAs, and 3562 (23.6%) end-of-day diary surveys. Table 4 shows EMA and diary survey participation rates in terms of the mean days of reporting. The participants completed at least two surveys a day for 151.8 days (84%) on average, and the goal was 180 days.

**Table 4:** Ecological momentary assessments and diary reports of the participants, including participants with delayed 6-month follow-up assessments who continued the ecological momentary assessments and diary.

Characteristics	Mean (range)
Number of days it took to complete at least 1 survey	184.2 (126-242)
Number of days it took to complete at least 2 surveys	149.3 (18-230)
Number of days it took to complete at least 4 surveys	47.8 (10-169)

**Table 5:** Participation rates for ecological momentary assessments and diary surveys, recall self-reports, and biomarker assessments (n=29).

Characteristics		Participants, n (%)
**Recall self-reports**		
	Number of follow-up assessment meetings	58^a^	
	Physical activity	48 (82.8)
	Intake of fruits and vegetables	50 (86.2)
	Intake of food with high-sugar content	47 (81.0)
	Intake of fast food	47 (81.0)
**Biomarker assessments**		
	Body fat and body mass index and blood pressure	58 (100.0)
	Epstein–Barr virus and C-reactive protein level	54 (93.1)

^a^Number of follow-ups that were completed by participants (29 participants who completed two follow-ups each).

As shown in Table 5, the participants completed 58 follow-ups (29 participants × 2 follow-ups). The completion rates for the individual recall and biomarker measures were fairly high on average (range 81.0%-100% of the recall or biomarker measures).

### Associations Between Ecological Momentary Assessment and Diary Reports and Recall and Biomarker Data

Table 1 presents the bivariate associations across domains (diet, stress, and physical activity) between the pairs of EMA and diary measures and either a recall or biomarker measure. Considering the large number of possible pairs, Table 1 presents only the associations between EMA and diary measures and alternative measures of the same domain (eg, recalls) or the different domains if the *P* value was ≤.10 for the association. Small-to-moderate correlations were observed.

Diet quality EMA and daily diary ratings were both negatively associated with the recall of the intake of food with high-sugar content (r=−.52, *P*<.01 and r=−.40, *P*=.04, respectively). Diet quality EMA ratings, but not those of daily diary, were positively associated with the intake of fruits and vegetables (r=.34, *P*<.01) and moderate physical activity in terms of minutes per day (r=48, *P*=.03) and negatively associated with the intake of fast food (r=−.42, *P*=.02), systolic blood pressure (r=−.32, *P*=.02), and C-reactive protein levels (r=−.34, *P*=.02). Ratings of the diet diary were also associated with vigorous physical activity in terms of minutes per day (r=.47, *P*=.02) and total minutes (r=.35, *P*<.01). Based on both R^2^ statistics, roughly a quarter of the variance in the relationship between diet EMA reports and high-sugar counts was explained (R^2^_Ed_=.25 and R^2^_Total_=.28). Based on the R^2^_Ed_ statistics, a similar level of variability was found for the relationship between reports on diet diary and vigorous physical activity in terms of minutes per day (R^2^=.34) and total minutes (R^2^=.45). A low level of variability was observed for the remaining relationships between diet measures with recall reports and biomarkers.

Daily stress diary reports correlated with the PSM-9 recall or global measure had the highest correlation among the results (r=.50, *P*=.02) and had trends in correlations with walking activity recalls. Stress EMA was also correlated with PSM-9 (r=.30, *P*=.01) and recall for walking minutes per day (r=.27, *P*=.05). Similar to the dietary report, the R^2^_Ed_ statistics for stress EMA reports (R^2^_Ed_=.27) and both R^2^statistics for stress daily reports (R^2^_Ed_=.28 and R^2^_Total_=.24) indicate that approximately a quarter of the variation was caused by the relationship with PSM-9 recall.

Daily diary physical activity reports were not significantly associated with their corresponding recall measures, whether counted by days, minutes per day, or total minutes. Light physical activity (counted as days) was associated with moderate activity recall days (r=.43, *P*=.04). Moderate physical activity days were negatively associated with BMI (r=−.40, *P*=.02). Vigorous physical activity days were negatively associated with BMI and body fat (r=−.44, *P*=.02 for both measures). Based on the R^2^_Ed_ statistics, a quarter of the variance for physical activity days was explained by the relationships with BMI (R^2^_Ed_=.26) and body fat (R^2^_Ed_=.25). The variance was fairly low for the remaining relationships between physical activity reports and both recall reports and biomarkers.

**Table 6:** Final random effects linear regression models showing the associations between ecological momentary assessment and diary and recall-based predictor variables.

Ecological momentary assessment or diary response predictor		n	β^a^	SE	R^2^_Ed_^b^	R^2^_Total_^c^
Diet ecological momentary assessment (3 times per day): Intake of high-sugar food^d^		34	−0.0055	0.0018	0.27	0.24
**Diet diary: Model 1**		29	—^e^	—	0.39	0.29
	Intake of high-sugar food^f^	—	−0.011	0.0051	—	—
	Vigorous physical activity (min/day)^f^	—	0.0071	0.0026	—	—
**Diet diary: Model 2**		28	—	—	0.45	0.23
	Intake of high-sugar food^f^	—	−0.011	0.0054	—	—
	Vigorous physical activity (total min)^d^	—	0.0021	0.00066	—	—
Stress ecological momentary assessment (3 times per day): PSM-9^g^ (stress inventory)^f^		45	0.014	0.0050	0.27	0.08
Stress diary: PSM-9 (stress inventory)^f^		31	0.024	0.0092	0.28	0.24

^a^β: regression coefficients.

^b^Based on the calculation of Edwards et al (2008).

^c^Based on ratio of unexplained-to-total variance.

^d^*P* value<.01.

^e^Not applicable.

^f^*P* value<.05.

^h^PSM-9: nine-item psychological stress measure.

### Multivariate Regression Analyses

Table 6 shows the multivariate regression results for EMA and diary measures that were significantly associated with a recall or biomarker measure as well as the direct corresponding recall measures (ie, variables shown in Table 1). Results indicated that diet EMA is most strongly associated with high-sugar food recalls, indicating the particular sensitivity for low-quality foods. The associations between stress EMA and diary reports as well as their corresponding PSM-9 recall were confirmed in the multivariate models and washing out associations with low physical activity indicated in the bivariate models. The only multivariate model with multiple significant predictors was the diet diary rating in which both high-sugar food and vigorous physical activity (total minutes and minutes per day) recalls retained their significant associations and together explained almost half of the variability diet diary rating based on the R^2^ measures.

## Discussion

### Principal Findings

The results in this analysis present several sets of inferences on the reliability and validity of the brief EMA and daily diary reports on diet, stress, and physical activity. Intermethod reliability between EMA and diary reports and their corresponding recall reports is moderate for stress and diet, as hypothesized. However, it was unexpectedly low for physical activity. In contrast to intermethod reliability, concurrent validity with other measures was demonstrated for diet and physical activity EMA and diary reports. However, it was not observed for stress.

Diet EMA as simple and subjective as “high or medium or low quality” ratings showed reliability with brief food frequency questionnaire recall methods and concurrent validity with physical activity. This is remarkable considering the subjective quality of the question and response options. As hypothesized, EMA appears to be more reliable and valid compared with the end-of-day diaries, particularly for food quality reports, and to some key diet- or systemic-related biomarkers. By contrast, daily diary diet ratings are not associated with the biomarkers and are more significantly associated with vigorous activity recalls than food count recalls. Daily diary diet reports are likely associated with a combination of recall and rounding errors that reflect the linked lifestyle habits of healthy eating and regular physical activity or poor diet and sedentary behaviors.

The simple stress self-monitoring questions (both EMA and daily diary) used in this study also indicated good intermethod reliability with the PSM-9 brief recall measure. The trends in the associations between stress EMA and diary as well as walking recall report data demand further explanation and analysis. Qualitative reports from formative work (BLINDED) have identified that mothers have lower levels of physical activity due to stress, specifically, time pressures associated with stress result in the lack of time to engage in moderate or vigorous physical activity. Walking as physical activity in daily life (eg, for transport and walking pets) may be more salient for reportings when stressed and unable to engage in intentional moderate or vigorous physical activity.

The moderate negative associations between vigorous physical activity diary reports and BMI and body fat and similar trends in the associations with moderate physical activity indicate the concurrent validity of the physical activity diary reports. Vigorous physical activity reports likely reflect the classes of highly active (or inactive) participants who are consistent enough in their activity levels to observe the associations with biomarkers. Notably, the crudest calculations of physical activity (ie, days vs minutes for vigorous and moderate physical activity) show potential trends for intermethod reliability correlations with their corresponding recall reports, whereas light physical activity diary reports showed significant correlations with moderate activity.

Concurrent validity with different biomarkers and biomeasures for different activity measurement methods also suggests that the underlying aspects of physical activity measured using different methods may be somewhat independent [17]. Similar challenges were observed for food frequency questionnaires [18] and are reported to vary in terms of individuals and population groups [56-58].

### Limitations

The primary limitation of this study is its relatively small sample size. A larger sample size would increase statistical power, which would likely result in trends showing statistical significance (ie, *P*=.10) and more elaboration between variables in the multivariate analyses. Another limitation is the participant-centered design that prioritized brief recall measures and salient EMA and diary question design over highly detailed measures that might have better reliability and validity but higher user burden and lower engagement, sustainability, and completion rates. There are additional limitations in evaluating the magnitude of the associations between measures. Given that some study measures, such as diet quality, measure of the self-perceptions of health, the degree of association between self-reported measures likely includes method bias; for example, a high perception of health may lead to positive reports on both diet quality and physical activity. Finally, the 6-month study period is in accordance with the time periods for weight loss interventions and other lifestyle modification programs; 6 months is a long period for an EMA study and calls for additional research to determine optimal EMA schedules that compliment lifestyle modification program schedules.

### Conclusions

The results of this study suggest that simple and brief EMA measures for diet and daily diary measures for stress, may be good enough tools for long periods of self-monitoring. This is especially important in the study population that mostly includes ethnic minority mothers, several of whom expressed that time is a barrier in monitoring and engaging in healthy behaviors. The inconsistencies between self-report and objective measurement methods and the lack of gold standards have resulted in recommendations to use a combination of methods, particularly when examining impacts on health status [17,59,60]. Future studies with larger sample sizes must be conducted while examining active and passive self-monitoring strategies and ecological momentary interventions [61] that trigger microinterventions based on the self-monitoring and contextual data (eg, global positioning system location) of smartphone apps [62,63]. There are significant challenges in addressing this in future studies. The intersecting issues of burden, participation (compliance), and timelines for changing and then sustaining daily health routines must be carefully considered. Initially, intensive EMA and diary self-reporting could support changes in behaviors. Once improvement in health status is achieved (eg, weight loss and improved cardiovascular fitness), the next hurdle is to maintain positive outcomes by continuously performing behavioral routines. Apps must be adaptive to the stages of change, participation burnout, and varying patterns of setbacks that individuals experience in adopting and maintaining healthier routines. However, smartphone apps are well suited for the task.

## Abbreviations

BMI: body mass index
CHIS: California Health Interview Survey
ELISA: enzyme-linked immunosorbent assay
EMA: ecological momentary assessment
